# The Infertility-Related Stress Scale: Validation of a Brazilian–Portuguese Version and Measurement Invariance Across Brazil and Italy

**DOI:** 10.3389/fpsyg.2021.784222

**Published:** 2022-01-13

**Authors:** Giulia Casu, Victor Zaia, Erik Montagna, Antonio de Padua Serafim, Bianca Bianco, Caio Parente Barbosa, Paola Gremigni

**Affiliations:** ^1^Department of Psychology, University of Bologna, Bologna, Italy; ^2^Postgraduate Program in Health Sciences, Centro Universitário FMABC, Santo André, Brazil; ^3^Ideia Fértil Institute of Reproductive Health, Centro Universitário FMABC, Santo André, Brazil; ^4^Neuropsychology Unit, Department and Institute of Psychiatry, University of São Paulo School of Medicine, São Paulo, Brazil; ^5^Department of Psychology, Methodist University of São Paulo, São Bernardo do Campo, Brazil

**Keywords:** infertility-related stress, validation, exploratory structural equation modeling, bifactor model, measurement invariance, depressive symptoms

## Abstract

Infertility constitutes an essential source of stress in the individual and couple’s life. The Infertility-Related Stress Scale (IRSS) is of clinical interest for exploring infertility-related stress affecting the intrapersonal and interpersonal domains of infertile individuals’ lives. In the present study, the IRSS was translated into Brazilian–Portuguese, and its factor structure, reliability, and relations to sociodemographic and infertility-related characteristics and depression were examined. A sample of 553 Brazilian infertile individuals (54.2% female, mean aged 36 ± 6 years) completed the Brazilian–Portuguese IRSS (IRSS-BP), and a subsample of 222 participants also completed the BDI-II. A sample of 526 Italian infertile individuals (54.2% female, mean aged 38 ± 6 years) was used to test for the IRSS measurement invariance across Brazil and Italy. Results of exploratory structural equation modeling (ESEM) indicated that a bifactor solution best represented the structure underlying the IRSS-BP. Both the general and the two specific intrapersonal and interpersonal IRSS-BP factors showed satisfactory levels of composite reliability. The bifactor ESEM solution replicated well across countries. As evidence of relations to other variables, female gender, a longer duration of infertility, and higher depression were associated with higher scores in global and domain-specific infertility-related stress. The findings offer initial evidence of validity and reliability of the IRSS-BP, which could be used by fertility clinic staff to rapidly identify patients who need support to deal with the stressful impact of infertility in the intrapersonal and interpersonal life domains, as recommended by international guidelines for routine psychosocial care in infertility settings.

## Introduction

According to the World Health Organization (World Health Organization [WHO], 2006), approximately 10% of couples of reproductive age worldwide have difficulties achieving pregnancy. Infertility has been defined as the absence of conception after 12 months of regular unprotected sexual intercourse (Zegers-Hochschild et al., 2017). It is estimated that more than 48 million couples worldwide suffer from infertility (Mascarenhas et al., 2012), and most of them live in developing countries (Ombelet et al., 2008). In Brazil, it is estimated that 15–20% of couples of reproductive age have some infertility problems (Instituto Brasileiro de Geografia e Estatística [IBGE], 2010).

Infertile individuals and couples experience considerable stress because of failing to achieve a meaningful life goal such as parenthood and its accompanying social stigma (Loke et al., 2012; Öztürk et al., 2021a). Stress can affect immune system activity and thus lead to physical and mental vulnerability (Segerstrom and Miller, 2004). Implications of infertility include physical symptoms, reduced psychological well-being and quality of life, feelings of guilt and shame, use of negative avoidance coping strategies, and social isolation (Rockliff et al., 2014; Luk and Loke, 2015; Rooney and Domar, 2016; Swanson and Braverman, 2021). Recently, a prevalence of 21–52% has been reported for depressive symptoms among infertile women, which is well above rates in the general population (Kiani et al., 2021). Women are the most affected by the infertile experience, consistently showing greater social vulnerability, stress and emotional distress, and lower quality of life than men (Patel et al., 2018; Casu et al., 2019; Ha and Ban, 2020).

To solve their infertility problems, more than half of infertile couples seek medical care like assisted reproductive technology (ART) treatments (Boivin et al., 2007). ART treatments constitute an additional source of stress for infertile couples due to expensive, time-consuming, invasive, and physically demanding procedures, along with the uncertainty of the outcomes (Öztürk et al., 2021b). Indeed, infertile patients, and especially women, report that ART treatments are physically and emotionally exhausting (Arya and Dibb, 2016; Anaman-Torgbor et al., 2021; Öztürk et al., 2021b), which is a major cause of premature treatment discontinuation by 30% of couples (Pedro et al., 2017; Domar et al., 2018). Altogether, the specific stress associated with infertility and its treatment can have long-lasting psychosocial consequences on infertile individuals and couples (Schmidt, 2009).

The stress specific to infertility, namely infertility-related stress, has been conceptualized as the burden that the infertile condition imposes on different life domains and entails areas of patient concern such as social, relationship, and sexual concerns, need for parenthood, and negative evaluation of childlessness (Newton et al., 1999; Schmidt et al., 2005). Thus, infertility-related stress has effects at the individual intrapersonal and interpersonal levels. Screening infertile patients for their levels of infertility-related stress can be especially useful in ART settings. Indeed, identifying patient needs by fertility staff has been recommended by the European Society of Human Reproduction and Embryology (ESHRE) guidelines for routine psychosocial care to reduce stress and improve patient well-being and compliance with treatment (Gameiro et al., 2015).

Various measures have been developed to assess infertility-related stress, such as the 46-item Fertility Problem Inventory (FPI; Newton et al., 1999) and the 36-item Fertility Quality of Life Tool (FertiQoL; Boivin et al., 2011). However, time constraints in health settings, such as fertility clinics, make the use of brief measures advisable to minimize patient and staff burden and provide a time-efficient assessment (Ziegler et al., 2014). A brief self-report questionnaire has been recently developed to measure intrapersonal and interpersonal infertility-related stress in infertile Italian individuals, namely the Infertility-Related Stress Scale (IRSS) (Casu and Gremigni, 2016). The IRSS intrapersonal dimension refers to one’s identity and resources for mind and body well-being as affected by infertility stress and includes aspects such as mental and physical health, intimacy, leisure, and life satisfaction. The interpersonal dimension refers to one’s social roles, rights, and responsibilities and includes aspects like relationships with others in the social and familial environment and work performance. In the original validation study on a sample of 597 infertile women and men turning to ART (Casu and Gremigni, 2016), the IRSS showed evidence of good psychometric properties. Confirmatory factor analysis (CFA) supported the proposed two-correlated factor model of infertility-related stress impacting intrapersonal and interpersonal life domains. The two latent variables were strongly correlated (*r* = 0.72). The intrapersonal and interpersonal dimensions showed adequate internal consistency (Cronbach’s αs of 0.89 and 0.87, respectively) and test-retest reliability over 4 weeks (intraclass correlation coefficients of 0.89 and 0.86, respectively). As for evidence of relations to other variables, in both women and men, the intrapersonal dimension was strongly (*r*s between 0.43 and 0.55), and the interpersonal dimension moderately (*r*s between 0.29 and 0.40) correlated with measures of anxiety and depression. Also, infertility-related stress was higher in the intrapersonal than in the interpersonal area of life in both genders, and women scored strongly higher than men in the intrapersonal domain (Casu and Gremigni, 2016).

Therefore, the IRSS appears to be a promising tool that might assist fertility staff in screening for infertility-related stress and identifying the areas of life most disrupted by the infertile condition. The IRSS has been used previously in the Brazilian research context, showing high levels of internal consistency for its global score, which correlated negatively with spirituality, quality of life, and perceived social support scores, and positively with avoidance coping scores (Casu et al., 2018, 2019). However, a Brazilian–Portuguese version has not been rigorously validated yet, and it is unclear whether its factor structure reflects a cross-cultural pattern (Casu and Gremigni, 2016).

This study aimed to validate a Brazilian–Portuguese translation of the IRSS (IRSS-BP) by testing for its factor structure, reliability, and relations to sociodemographic and infertility-related characteristics and depression as evidence of concurrent validity. To investigate the IRSS-BP factor structure, exploratory structural equation modeling (ESEM; Asparouhov and Muthén, 2009) was preferred over CFA. CFA unrealistically assumes that each item represents its designated construct exclusively, forcing items to load on only one factor and constraining all cross-loadings to zero (Marsh et al., 2014). To overcome the limitations of CFA, ESEM has been recommended, which combines the flexibility of exploratory factor analysis with the advantages of CFA (e.g., estimation of goodness-of-fit indices and the possibility of multigroup analysis) while offering a more realistic representation of the data (Morin et al., 2013; Marsh et al., 2014). Using ESEM, we tested both the two-correlated factor model proposed in the IRSS original validation study and a bifactor model with one general factor and two specific factors. Indeed, the strong correlation between the intrapersonal and interpersonal domains of infertility-related stress found in the original validation study may suggest the presence of a global factor underlying all IRSS indicators (Morin et al., 2016). Measurement invariance of the IRSS across Brazilian and Italian samples was also tested. Evidence of measurement invariance would indicate that Brazilian infertile individuals conceptualize and evaluate infertility-related stress similarly to Italian ones (Steenkamp and Baumgartner, 1998). Concerning sociodemographic characteristics considered in concurrent validity testing, previous studies reported that risk factors for higher infertility-related stress or lower psychological health included female gender (Ying et al., 2015), older age (Lakatos et al., 2017), and low educational level (Zaidouni et al., 2018). About infertility-related characteristics, suffering from primary infertility (i.e., no prior pregnancies), a longer duration of the infertility problem, and a diagnosis of female factor infertility were associated with higher infertility-related stress (Patel et al., 2016; Zaidouni et al., 2018). Concerning depression, infertility-related stress was found to significantly predict depressive symptoms among infertile women and men (Zurlo et al., 2020).

## Materials and Methods

### Participants and Procedure

After approval by the institutional review board at both Brazilian and Italian institutions, participants at both sites were invited to participate and explained the scope of the study. Participation was voluntary and anonymous. All participants provided informed consent, and the study was conducted following the Declaration of Helsinki.

Inclusion criteria for both Brazilian and Italian participants were being 18 years or older, able to fill out a questionnaire in Brazilian-Portuguese/Italian language without help, having a diagnosis of infertility defined as the failure to achieve a clinical pregnancy after 12 months or more of regular unprotected sexual intercourse (Zegers-Hochschild et al., 2017), and being currently in or seeking ART treatment.

Participants who met the inclusion criteria in Brazil were invited to complete a paper-and-pencil questionnaire which included the Brazilian–Portuguese version of the IRSS and items on sociodemographic (i.e., gender, age, education) and infertility-related characteristics. Infertility-related characteristics included duration of infertility (coded as 1–2, 3–4, 5–6, and >6 years), infertility type (i.e., primary infertility, defined as having never conceived despite at least 12 months of attempting conception, or secondary infertility, defined as having had at least one prior conception but being subsequently unable to conceive after at least 12 months of attempting conception), and infertility diagnosis (coded as no diagnosis, male factor, female factor, both male and female factor, and unexplained). A randomized subsample of participants also completed the Brazilian–Portuguese version of the Beck Depression Inventory-II (BDI-II; Beck et al., 1996; Gomes-Oliveira et al., 2012). Participants who met the inclusion criteria in Italy were invited to fill in the original Italian IRSS and asked questions on age, education, infertility type, and infertility diagnosis.

Brazilian participants were recruited at a fertility clinic in the São Paulo metropolitan region, Brazil. Italian participants were recruited at two private fertility clinics in the metropolitan area of Bologna, Italy.

### Measures

#### Infertility-Related Stress Scale

The IRSS (Casu and Gremigni, 2016) is a 12-item self-report measure to assess the amount of stress the infertility problem places on different aspects of life. It consists of two 6-item subscales referring to the intrapersonal (e.g., mental well-being) and interpersonal (e.g., friends) domains of life. Each item is rated on a 7-point scale from 1 (not at all) to 7 (a great deal). McDonald’s ω of the Italian IRSS in the present study (*n* = 526) was 0.89 for both the intrapersonal (95% CI 0.88–0.91) and interpersonal (95% CI 0.87–0.91) domains, and 0.93 (95%CI 0.92–0.94) for the total IRSS.

Two independent bilingual translators translated the Italian IRSS into Brazilian-Portuguese and then back-translated it into Italian. Discussion between the translators and the Italian-speaking researchers resolved any discrepancies between the original and back-translated versions. Semantic validation of the final Brazilian–Portuguese translation was then performed in two focus groups (Mayring, 2014) of infertile individuals (*n* = 6, 50% women, by group). All participants in the focus groups were undergoing infertility treatment and had different educational levels. The lowest educational level was 8 years of schooling (corresponding to compulsory education in Brazil), and the highest level was 21 years of schooling (corresponding to Ph.D.). Participants were asked for their opinion about the readability and clarity of the instrument. Only slight changes were proposed to enhance clarity of instructions, which were implemented by the second author who facilitated the focus groups. The final Brazilian-Portuguese version of the IRSS was reviewed and approved by all focus group participants and named IRSS-BP. The final IRSS-BP version did not show substantial differences from the initial one.

#### Beck Depression Inventory-II

The BDI-II (Beck et al., 1996) is a widely used 21-item self-report measure to assess cognitive, motivational, affective, and somatic symptoms of depression. For each item, respondents are asked to choose the statement that best describes their feelings in the past 2 weeks. Each item is scored 0–3, with higher scores indicating greater depression severity. We used the Brazilian–Portuguese validated version of the BDI-II (Gomes-Oliveira et al., 2012). A total score of >10 was considered to differentiate between participants with below- and above-threshold levels of depression (Gomes-Oliveira et al., 2012). McDonald’s ω in the present study (*n* = 222) was 0.89 (95% CI 0.87–0.91).

### Data Analysis

Prior to psychometric analyses, outliers and careless responders were identified and removed from the dataset to improve data quality (Curran, 2016). Multivariate outliers were defined as any case with a Mahalanobis distance (*D*^2^) above the critical χ^2^ value of 32.91 (12 degrees of freedom, *p* < 0.001). Careless responders were detected using the inter-item standard deviation (ISD) to measure an individual’s inconsistent responding. Participants with ISD values two standard deviations above the mean were considered careless responders (Marjanovic et al., 2015).

Preliminary analyses on the final dataset included item descriptive statistics, univariate and multivariate normality, and associations with sociodemographics (i.e., gender and age). At the univariate level, IRSS-BP items with skewness and kurtosis < |2| were considered normally distributed. To test for multivariate normality, the Henze-Zirkler test was used. Associations of IRSS-BP items with gender and age were examined using point-biserial and product-moment correlations, respectively.

To investigate the factor structure of the IRSS-BP, ESEM with target rotation (Asparouhov and Muthén, 2009) was conducted. Target rotation enables ESEM to be used in a confirmatory way by allowing for an *a priori* specified configuration of indicators for each factor. In addition to the principal loadings, all cross-loadings are freely estimated in target rotation but targeted to be as close to zero as possible (Asparouhov and Muthén, 2009). Two ESEM models were tested: a first-order model with two correlated factors and a bifactor model. In the two-correlated factor model, each item loaded on intrapersonal and interpersonal factors. In the bifactor model, a general factor (G-Factor) and two specific factors (S-Factor intrapersonal and S-Factor interpersonal) were included, and each item loaded directly and simultaneously on the G-Factor and both the S-Factors. We used oblique target rotation in the first-order model and orthogonal target rotation in the bifactor model (Reise, 2012). In both models, loadings ≥ 0.30 were considered relevant. Model parameters were estimated using the robust maximum likelihood method, robust to violations of multivariate normality, and recommended for variables with five or more response categories (Rhemtulla et al., 2012). The following goodness-of-fit indices were used: χ^2^, Satorra–Bentler scaled χ^2^ statistic (S-B χ^2^), root mean square error of approximation (RMSEA) and standardized root mean square residual (SRMR) ≤ 0.08, and comparative fit index (CFI) ≥ 0.95 (Hu and Bentler, 1999). In the case of non-optimal model fit, modification indices were examined to find the most parsimonious changes to the model to achieve an acceptable fit to the data. To identify the model to be retained, we considered parameter estimates (loadings and cross-loadings) in addition to model fit indices. According to Morin et al. (2016), the bifactor ESEM model should be preferred if the G-Factor and S-Factors are well-defined, and cross-loadings in the bifactor ESEM solution decrease compared to its first-order counterpart. McDonald’s (1970) omega (ω) coefficient of composite reliability was also considered, with values above 0.70 and 0.50 being satisfactory for measures corresponding to first-order and bifactor models, respectively (Perreira et al., 2018). For both models, we also considered the proportion of item variance explained by the model: σ^2^ error, σ^2^ true related to the first-order factors in the first-order ESEM and the G-Factor and S-Factors in the bifactor ESEM, and σ^2^ true related to cross-loadings (Perreira et al., 2018; Morin et al., 2020). For the bifactor model, the proportion of explained common variance (ECV) attributable to each factor was also computed (Reise et al., 2013).

Using data from the Brazilian and the Italian samples, a multigroup ESEM was conducted to test the measurement invariance of the most optimal model across the country. Increasingly restrictive models representing configural (invariant factor structure), metric/weak (invariant factor loadings), scalar/strong (invariant intercepts), strict (invariant uniqueness), latent variance-covariance matrix, and latent factor means invariance (Steenkamp and Baumgartner, 1998). Differences in fit between nested models were evaluated considering, in addition to the S-B χ^2^ difference test (ΔS-B χ^2^), changes in CFI (ΔCFI), RMSEA (ΔRMSEA), and SRMR (ΔSRMR). A ΔCFI ≤ 0.010 supplemented by a ΔRMSEA < 0.015 or a ΔSRMR < 0.010 were considered as indicative of a non-significant decrease in fit across models (Chen, 2007). If full measurement invariance did not hold, partial measurement invariance was considered by relaxing equality constraints on measurement parameters (Steenkamp and Baumgartner, 1998). The sample size of the Brazilian and Italian samples was established *a priori* as to have approximately 10 observations for each freely estimated parameter in the models (Kline, 2005).

Analysis of variance (ANOVA) was performed to examine cross-country differences in IRSS scores and to test for relations of the IRSS-BP with sociodemographics (i.e., gender and education), infertility-related characteristics (i.e., duration of infertility, infertility type, and infertility diagnosis), and depressive symptomatology levels. Repeated-measures ANOVA was used to investigate differences in scores across IRSS-BP dimensions. Pearson correlations were computed to test for the associations of age and BDI-II scores with IRSS-BP scores.

Interpretation of results was based on statistical significance (*p* < 0.05) and measures of effect size, with *r* of 0.10 considered small, 0.30 medium and 0.50 large, and *d* of 0.20 considered small, 0.50 medium and 0.80 large (Cohen, 1988). ESEM models were estimated using Mplus 8.4 (Muthén and Muthén, 1998–2017). All other analyses were performed with IBM SPSS 27 (SPSS Inc., Chicago, IL, United States).

## Results

### Detection of Outliers and Careless Responders

In Brazil, of 700 invited patients, 570 (81.4%) met the inclusion criteria and completed the IRSS-BP. Fourteen cases (2.5%) had a *D*^2^ above the critical χ^2^ value and were flagged as multivariate outliers. Three cases (0.5%) had both a *D*^2^ greater than the critical χ^2^ and an ISD value two standard deviations (*SD* = 0.66) above the mean (*M* = 1.08) and were flagged as both outliers and careless responders. The Brazilian sample used in subsequent analyses thus comprised *n* = 553 infertile patients. In Italy, of 680 invited patients, 557 (81.9%) met the inclusion criteria and completed the IRSS. Eleven cases (2.0%) were flagged as multivariate outliers due to *D*^2^ values greater than the critical χ^2^, 12 cases (2.2%) had ISD values two standard deviations (*SD* = 0.60) above the mean (*M* = 1.25) and were considered careless responders, and 8 additional cases (1.4%) were flagged as both outliers and careless responders. Therefore, the Italian sample used in the analyses included *n* = 526 infertile patients.

### Participants’ Characteristics

Brazilian participants (*n* = 553) were 54.2% female; most participants were highly educated, having a degree or post-degree, and had primary infertility. About one third had unexplained infertility, while 14.1% had not completed standard infertility evaluations and thus had not a specific infertility diagnosis yet. About age and education, women (*M* = 35.24, *SD* = 5.24, range 18–54 years) were slightly younger than men (*M* = 37.35, *SD* = 6.52, range 23–63 years) [*F*(1,548) = 17.72, *p* < 0.001, *d* = 0.36] and a slightly larger proportion of women (80.3%) than men (72.3%) were highly educated [χ^2^(1) = 4.48, *p* = 0.03]. There were no gender differences in duration of infertility [χ^2^(3) = 5.64, *p* = 0.13], type of infertility [χ^2^(1) = 1.38, *p* = 0.24], or infertility diagnosis [χ^2^(3) = 2.34, *p* = 0.51]. The subsample of 222 participants who also completed the BDI-II was 60.4% female (*n* = 134), mean aged 34.56 years (*SD* = 5.47, range 23–54), and 88.7% highly educated. There were no differences in infertility-related characteristics between Brazilian participants who completed only the IRSS-BP and those who also responded the BDI-II.

Italian participants (*n* = 526) were 54.2% female, and women (*M* = 36.20, *SD* = 4.62, range 23–50 years) were strongly younger than men (*M* = 40.36, *SD* = 6.23, range 25–59 years) [*F*(1,524) = 77.01, *p* < 0.001, *d* = 0.77]. No other statistically significant gender differences were found in the Italian sample.

Comparisons between the Brazilian and Italian samples showed that Brazilian participants were slightly younger than the Italians (*d* = 0.32), a larger proportion of Brazilian than Italian participants were highly educated, had primary infertility and had unexplained infertility. In contrast, a smaller proportion was undiagnosed or had both male and female factors. Characteristics of participants are presented in Table 1.

**TABLE 1:** Participants’ characteristics.

Characteristic	Brazilians (*n* = 553)	Italians (*n* = 526)	Comparison
Gender, women, *n* (%)	300 (54.2)	285 (54.2)	χ^2^(1) = 0.001
Age, years, mean (*SD*, range)	36.21 (5.95, 18–63)	38.10 (5.80, 23–59)	*F*(1,1074) = 28.01*
Education, high, *n* (%)	424 (76.7)	232 (44.1)	χ^2^(1) = 119.96*
Infertility type, primary, *n* (%)	375 (67.8)	283 (53.8)	χ^2^(1) = 22.24*
Infertility diagnosis, *n* (%)		–	χ^2^(4) = 82.45*
Undiagnosed	78 (14.1)^a^	110 (20.9)^b^	
Male	140 (25.3)^a^	142 (27.0)^a^	
Female	119 (21.5)^a^	99 (18.8)^a^	
Both male and female	53 (9.6)^a^	118 (22.4)^b^	
Unexplained	163 (29.5)^a^	57 (10.8)^b^	
**Infertility duration, *n* (%)**			
1–2 years	188 (34.0)	–	
3–4 years	165 (29.8)	–	
5–6 years	121 (21.9)	–	
> 6 years	79 (14.3)	–	

*Proportions with different superscript letters in the same row significantly differ at p < 0.05 (post hoc z-scores and Bonferroni correction).*

*p < 0.001.

### Preliminary Analyses of Brazilian–Portuguese Infertility-Related Stress Scale Items

All IRSS-BP items followed a univariate normal distribution, with skewness and kurtosis < |2|. However, the Henze-Zirkler test indicated significant departures from multivariate normality (HZ = 11.39, *p* = 0.008). As for relations to demographic variables (i.e., sex and age), associations with gender were significant for almost all items but only weak (*r*_*pb*_ = –0.21 to –0.07). Pearson’s correlations with age were predominantly non-significant and ranged between –0.11 and –0.01. Item descriptive statistics are shown in Supplementary Table S1.

### Factor Structure

The goodness of fit of the two-correlated factor solution was below acceptable levels [χ^2^(43) = 455.899, S-B χ^2^(43) = 332.264, *p* < 0.001, RMSEA = 0.110 (90% CI 0.099–0.122), SRMR = 0.034, CFI = 0.917]. Inspection of modification indices suggested that allowing the uniqueness of two items (i.e., item 2 – Relatives with item 3 – In-laws) covary would improve model fit. This covariation makes substantive and theoretical sense, as blood relatives and in-laws have been reported as the main sources of social pressure by infertile individuals (Hasanpoor-Azghdy et al., 2015). Therefore, the model was respecified, including an argument for the correlated uniqueness. The goodness of fit of the respecified model improved significantly, ΔS-B χ^2^(1) = 96.587, *p* < 0.001, with goodness-of-fit indices indicating acceptable fit to the data [χ^2^(42) = 264.564, S-B χ^2^(42) = 194.876, RMSEA = 0.081 (90% CI 0.070–0.093), SRMR = 0.027, CFI = 0.956]. As shown in Table 2, the two-correlated factor, first-order ESEM solution resulted in well-defined factors. Loadings on the target factor were > 0.40 for both the intrapersonal (*M*_|λ|_= 0.740) and intrapersonal (*M*_|λ|_= 0.773) dimensions. Loadings on non-target factors were significant and > |0.20| for 4 out of the 12 possible cross-loadings, ranging from 0.201 to 0.346. Composite reliability estimates were adequate for both factors (ω = 0.88). A correlation of 0.72 between the two factors was observed. Such a strong correlation might indicate the existence of a more general factor tapping variation in responses across all IRSS-BP items, supporting the estimation of a bifactor model.

**TABLE 2:** Standardized factor loadings and item uniquenesses for the first-order and bifactor ESEM solutions (*n* = 553).

	First-order ESEM			Bifactor ESEM			
	Intrapersonal (λ)	Interpersonal (λ)	δ	G-Factor (λ)	Intrapersonal (λ)	Interpersonal (λ)	δ
(1) Physical well-being	**0.714**	0.126	0.345	0.657	**0.466**	*0.031*	0.351
(4) Leisure and enjoyment	**0.445**	0.346	0.461	0.654	**0.302**	0.114	0.468
(5) Marital satisfaction	**0.617**	*0.106*	0.514	0.616	**0.341**	–0.132	0.486
(6) Mental well-being	**0.960**	–0.082	0.186	0.630	**0.656**	*0.013*	0.172
(9) Sexual pleasure	**0.752**	*0.037*	0.393	0.609	**0.477**	*–0.039*	0.400
(12) Global life satisfaction	**0.950**	–0.118	0.244	0.587	**0.650**	*0.004*	0.232
(2) Relatives	0.244	**0.589**	0.387	0.912	*–0.030*	**0.252**	0.104
(3) In-laws	0.201	**0.577**	0.460	0.837	*–0.017*	**0.179**	0.266
(7) Performance at work/housework	0.259	**0.597**	0.354	0.771	0.160	**0.159**	0.355
(8) Close friends	*–0.049*	**0.966**	0.131	0.878	*–0.019*	**0.292**	0.143
(10) Colleagues	–0.108	**0.995**	0.152	0.837	*–0.026*	**0.429**	0.114
(11) Neighbors	–0.152	**0.914**	0.341	0.734	–0.066	**0.332**	0.347
ECVf				75.8%	17.6%	5.8%	
ECVc					0.4%	0.4%	
ω	0.88	0.88		0.96	0.75	0.53	

*ESEM, exploratory structural equation model; λ, factor loading; δ, uniqueness; ECVf, explained common variance of factors; ECVc, explained common variance of cross-loadings; ω, omega coefficient of composite reliability; target factor loadings on the specific factors are in bold; non-significant parameters (p ≥ 0.05) are in italics.*

The goodness of fit of the bifactor ESEM solution was adequate, with all goodness-of-fit indices meeting the pre-established criteria [χ^2^(33) = 201.028, S-B χ^2^(33) = 150.380, *p* < 0.001, RMSEA = 0.080 (90% CI 0.067–0.093), SRMR = 0.022, CFI = 0.966]. Inspection of parameter estimates (Table 2) indicated that the G-Factor was well-defined, with strong and positive factor loadings in all IRSS-BP items, ranging between 0.587 and 0.912 (*M*_|λ|_= 0.727). Loadings of the G-Factor ranged from 0.587 to 0.657 for the intrapersonal S-Factor (*M*_|λ|_= 0.626) and from 0.734 to 0.912 for the interpersonal S-Factor (*M*_|λ|_= 0.828). Composite reliability estimate indicated that the G-Factor was highly reliable (ω = 0.96). The G-Factor explained about 76% of the common variance extracted. The intrapersonal S-Factor was well-defined, with significant and relevant loadings for all its items (|λ|= 0.302 to 0.650, *M*_|λ|_= 0.482). Two items (item 6 and 12) loaded higher on this S-Factor than on the G-Factor, with small differences in loadings (Δλ = 0.026 and 0.063 for item 6 and item 12, respectively). The remaining four items loaded more strongly on the G-Factor than the S-Factor, with differences in loadings between 0.132 (item 9) and 0.352 (item 4). The intrapersonal S-Factor showed satisfactory reliability (ω = 0.75) and explained about 18% of the variance in the items. The interpersonal S-Factor was relatively well defined (|λ|= 0.159–0.429, *M*_|λ|_= 0.274), as four target items (items 2, 3, 7, and 8) had loadings lower than 0.30 (although just below 0.30 for item 8), indicating that the variance in these items was primarily used in defining the G-Factor, with differences in loadings ranging from 0.586 (item 8) to 0.660 (item 2). The remaining two items had relevant loadings but loaded higher on the G-Factor than the S-Factor (Δλ = 0.408 for item 10 and 0.402 for item 12). The reliability level was acceptable (ω = 0.53), and this S-Factor explained about 6% of the common variance.

Comparison of factor loadings between the two ESEM solutions showed that the cross-loadings ranged from 0.037 to 0.346 (*M*_|λ|_= 0.152) in the two-correlated factor ESEM solution, and from 0.004 to 0.160 (*M*_|λ|_= 0.054) in the bifactor ESEM solution (Table 2). No cross-loadings > |0.20| remained in the bifactor ESEM solution compared with the first-order ESEM solution, probably due to the inclusion of the G-Factor in the model. The ECV assumed by the cross-loadings in the bifactor ESEM solution was only 0.8%. The average proportion of variance in the items explained by the factors (σ^2^ true total) was 63.7% in the two-correlated factor ESEM solution and 71.3% in the bifactor ESEM solution (Supplementary Table S2).

Overall, these results support the superiority of the bifactor model, which was retained for subsequent analyses of measurement invariance.

### Measurement Invariance and Differences in Infertility-Related Stress Scale Scores Across Brazil and Italy

In the tests of measurement invariance across countries (Table 3), invariance of factor structure (configural), factor loadings (metric/weak), and item intercepts (scalar/strong) across Brazil and Italy was supported. Although the ΔS-B χ^2^ tests were statistically significant, changes in CFI, RMSEA, and SRMR values remained acceptable across nested models. Full strict invariance was not achieved. Based on both modification indices for the strict invariance model and parameter estimates of the scalar/strong invariance model, we allowed the uniqueness of item 2 (Relatives) to be freely estimated across countries. The uniqueness of this item was 0.103 in the Brazilian sample and 0.302 in the Italian sample. The model of strict partial invariance was supported and retained in subsequent invariance tests. The models including equality constraints on the latent variance-covariance matrix and the latent factor means did not substantially decrease model fit, indicating invariant latent variance-covariance and factor means across Brazilian and Italian infertile individuals.

**TABLE 3:** Goodness-of-fit indices for tests of measurement invariance (*n* = 1,079).

Level of invariance	*df*	S-B χ^2^	Δ*df*	ΔS-B χ^2^	CFI	ΔCFI	RMSEA	ΔRMSEA	SRMR	ΔSRMR
Configural	66	296.899*	–	–	0.964	–	0.081	–	0.022	–
Metric/weak	93	333.615**	27	50.211*	0.962	–0.002	0.069	–0.012	0.037	+0.015
Scalar/strong	102	422.639*	9	107.493**	0.950	–0.012	0.076	+0.007	0.041	+0.004
Strict	114	550.531**	12	151.742**	0.932	–0.018	0.084	+0.008	0.042	+0.001
Strict partial	113	476.290**	11	51.076*	0.943	−0.007	0.077	+0.001	0.042	+0.001
Latent variance-covariance	119	482.148**	6	8.044	0.943	0.000	0.075	–0.002	0.049	+0.007
Latent means	122	484.220**	3	1.134	0.943	0.000	0.074	–0.001	0.051	+0.002

*Brazil: n = 553 (51.3%); Italy: n = 526 (48.7%); in the partial strict invariance model, uniqueness of item 2 was freely estimated across countries.*

*p < 0.01, **p < 0.001.

No significant differences between Brazilian and Italian participants were found in the observed scores for the G-Factor (Brazil: *M* = 32.08, *SD* = 16.54; Italy: *M* = 33.00, *SD* = 15.54) [*F*(1,1077) = 0.89, *p* = 0.35, *d* = 0.06], intrapersonal S-Factor (Brazil: *M* = 17.57, *SD* = 9.06; Italy: *M* = 18.32, *SD* = 8.53) [*F*(1,1077) = 1.95, *p* = 0.16, *d* = 0.09] nor in the interpersonal S-Factor (Brazil: *M* = 14.51, *SD* = 8.73; Italy: *M* = 14.68, *SD* = 8.13) [*F*(1,1088) = 0.12, *p* = 0.73, *d* = 0.02].

### Relations to Sociodemographic and Infertility-Related Characteristics and Depressive Symptomatology

Associations with sociodemographic and infertility-related characteristics and depression were calculated only in the Brazilian sample to test for the IRSS-BP concurrent validity. Age was unrelated to IRSS-BP overall (*r* = –0.06, *p* = 0.17), intrapersonal (*r* = –0.08, *p* = 0.06) and interpersonal domain scores (*r* = –0.03, *p* = 0.51). Results of group comparisons and descriptive statistics are displayed in Table 4. Interactions between sociodemographic and infertility-related characteristics were non-significant. Gender and duration of infertility had significant main effects on global IRSS-BP and both IRSS-BP domains. Compared to men, women reported slightly higher mean scores on global IRSS-BP scores (*d* = 0.36) as well as on both the intrapersonal (*d* = 0.36) and interpersonal (*d* = 0.30) domains. Participants who had been trying to conceive for 1–2 years reported lower scores than participants who had been trying for 3–4, 5–6, and >6 years in the global IRSS-BP (*d* = 0.40–0.50) and in the intrapersonal (*d* = 0.27–0.35) and interpersonal (*d* = 0.42–0.58) IRSS-BP domains. In the intrapersonal domain, the levels of stress reported by participants who had been trying to conceive for 1–2 years and >6 years did not differ significantly.

**TABLE 4:** Comparisons between groups in IRSS-BP scores (*n* = 553).

	G-Factor			Intrapersonal domain			Interpersonal domain			
	*M*	*SD*	Effect	*M*	*SD*	Univariate effect	*M*	*SD*	Univariate effect	Multivariate effect
Gender			*F*(1,416) = 9.27**			*F*(1,416) = 9.66**			*F*(1,416) = 6.15*	Wilks’ λ = 0.98 *F*(2,415) = 4.92**
Women (*n* = 300)	34.75	16.75		19.06	9.18		15.69	8.88		
Men (*n* = 253)	28.91	15.74		15.81	8.62		13.10	8.34		
Education			*F*(1,416) = 0.06			*F*(1,416) = 0.04			*F*(1,416) = 0.06	Wilks’ λ = 1.00 *F*(2,415) = 0.03
Up to high school (*n* = 129)	32.26	17.92		17.51	9.46		14.75	9.55		
Degree/post-degree (*n* = 424)	32.02	16.12		17.59	8.95		14.43	8.47		
Duration of infertility			*F*(3,416) = 3.70*			*F*(3,416) = 3.28*			*F*(3,416) = 3.18*	Wilks’ λ = 0.97 *F*(6,830) = 2.16*
1—2 years (*n* = 188)	27.66^a^	13.72		15.74^a^	8.31		11.92^a^	6.96		
3–4 years (*n* = 165)	33.87^b^	17.20		18.56^b^	9.20		15.30^b^	9.01		
5-6 years (*n* = 121)	35.31^b^	17.36		18.77^b^	9.43		16.54^b^	9.27		
>6 years (*n* = 79)	33.92^b^	18.05		18.03^ab^	9.43		15.90^b^	9.69		
Infertility type			*F*(1,416) = 1.19			*F*(1,416) = 2.14			*F*(1,416) = 0.27	Wilks’ λ = 0.99 *F*(2,415) = 1.30
Primary (*n* = 375)	32.13	16.19		17.49	8.97		14.65	8.57		
Secondary (*n* = 178)	31.97	17.31		17.75	9.29		14.22	9.06		
Infertility diagnosis			*F*(4,416) = 2.55			*F*(4,416) = 2.35			*F*(4,416) = 2.24	Wilks’ λ = 0.97 *F*(8,830) = 1.71
Undiagnosed (*n* = 78)	34.12	16.95		18.87	9.46		15.24	8.63		
Male (*n* = 140)	29.68	13.87		16.31	7.77		13.36	7.57		
Female (*n* = 119)	33.08	16.98		18.40	9.38		14.68	8.90		
Both male female (*n* = 53)	30.09	16.90		16.40	9.67		13.70	8.57		
Unexplained (*n* = 163)	33.08	17.85		17.80	9.39		15.28	9.56		
BDI-II (*n* = 222)			*F*(1,218) = 21.73***			*F*(1,218) = 30.16***			*F*(1,218) = 9.48**	Wilks’ λ = 0.88 *F*(2,217) = 15.48***
Below-threshols (*n* = 148)	30.78	15.74		16.51	8.71		14.27	8.43		
Above-threshold (*n* = 74)	42.38	16.41		23.78	8.52		18.59	9.10		

*Score range was 12–84 for global IRSS-BP (G-Factor) and 6–42 for intrapersonal and interpersonal domains (S-Factors). Means with different superscript letters in the same column significantly differ at p < 0.05 (Bonferroni post hoc multiple comparisons).*

*p < 0.05, **p < 0.01, ***p < 0.001.

**FIGURE 1:**
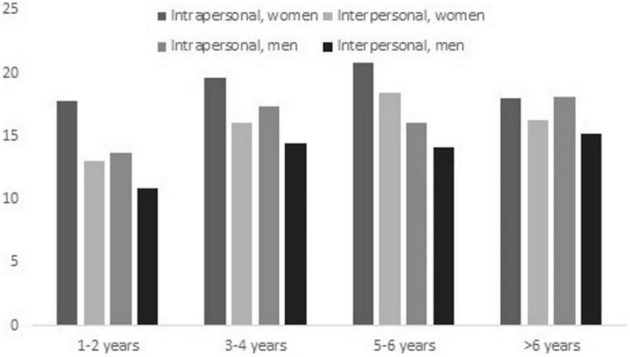
Brazilian–Portuguese IRSS domains by gender and duration of infertility.

Repeated measures ANOVA was used to detect whether the perceived impact of infertility-related stress was greater in one of the IRSS-BP domains than in the other. Regardless of gender and duration of infertility, mean scores in the intrapersonal domain (*M* = 17.57, *SD* = 9.06) were significantly higher than scores in the interpersonal domain (*M* = 14.51, *SD* = 8.73) [*F*(1,545) = 93.41, *p* < 0.001, *d* = 0.34]. As shown in Figure 1, differences between IRSS-BP domains were primarily related to the duration of infertility. While the effect size of the difference between intrapersonal and interpersonal infertility-related stress was small in both women (*d* = 0.37) and men (*d* = 0.32), it was medium for a duration of infertility of 1–2 years (*d* = 0.50) and small for more than 2 years of attempting to conceive (*d* = 0.22–36).

In the subsample of participants who also completed the BDI-II (*n* = 222), 66.7% reported below-threshold and 33.3% above-threshold levels of depressive symptoms. Regardless of gender, participants with above-threshold BDI-II scores reported significantly higher scores in all IRSS-BP factors than those with below-threshold BDI-II scores (Table 4). The effect size of these group differences was medium-to-large for global IRSS-BP (*d* = 0.73), large for the intrapersonal domain (*d* = 0.84), and medium for the interpersonal domain (*d* = 0.50).

Bivariate correlations with BDI-II scores in the total subsample were *r* = 0.38 for global IRSS-BP, *r* = 0.43 for the intrapersonal domain, and *r* = 0.26 for the interpersonal domain (*p* < 0.001). Computation of correlations by gender indicated that associations with BDI-II scores among men (*n* = 88) were non-significant for global IRSS-BP (*r* = 0.18, *p* = 0.10) and the interpersonal domain (*r* = 0.08, *p* = 0.10), and significant and small-to-moderate for the intrapersonal domain (*r* = 0.24, *p* = 0.02). Among women (*n* = 134), correlations with BDI-II scores were significant and strong for total IRSS-BP and the intrapersonal domain (*r* = 0.48 and 0.53, respectively, *p* < 0.001) and moderate for the interpersonal domain (*r* = 0.35, *p* < 0.001).

## Discussion

The present study aimed to assess the IRSS psychometric properties in its Brazilian–Portuguese language version (IRSS-BP). We examined the factor structure, reliability, and relations to other variables of the IRSS-BP on a sample of Brazilian infertile individuals who were undergoing or seeking ART treatment. We also used a sample of infertile Italian individuals to test for measurement invariance across Brazil and Italy, as the IRSS was initially developed and validated in Italy (Casu and Gremigni, 2016).

Exploratory structural equation modeling (ESEM) with target rotation (Asparouhov and Muthén, 2009) was used in this study to investigate the IRSS-BP factor structure. We tested both the two-correlated factor model proposed in the IRSS original validation study and a bifactor model where each item simultaneously loaded on a general factor (G-Factor) and two specific factors (S-Factor intrapersonal and S-Factor interpersonal). The goodness of fit of the original first-order factor model was acceptable after allowing covariation between the uniqueness of two items. The two factors were strongly related, showing the same correlation of 0.72 reported in the IRSS original validation study (Casu and Gremigni, 2016). Noteworthy, such a strong correlation between the first-order factors supported the specification and testing of an alternative bifactor model, including the presence of an underlying global construct (Morin et al., 2016). The bifactor ESEM solution had an adequate fit to the data. Inspection of item loadings revealed a highly reliable G-Factor defined by positive and strong loadings for all items. All IRSS-BP items except two loaded more strongly on the G-Factor than the respective S-Factors. The G-Factor explained 76% of the common variance in the items, with the remaining 24% spread across the S-Factors. All target loadings on the intrapersonal S-Factor were significant and relevant, indicating that this S-Factor reflects meaningful specificity not represented in the G-Factor (Morin et al., 2016). Therefore, the intrapersonal S-Factor was well-defined and showed a good ω reliability estimate. The interpersonal S-Factor was less well defined, as target loadings were all significant but non-relevant for three items and just below 0.30 for one item. This indicates that the interpersonal S-Factor maintained limited specificity once the G-Factor was considered. However, the composite reliability estimate was above acceptable levels (i.e., >0.50) indicating that scores in the interpersonal S-Factor accounted for a non-negligible amount of variation beyond the G-Factor (Perreira et al., 2018).

Comparison of ESEM solutions revealed that no cross-loadings > |0.20| remained in the bifactor solution compared with the first-order solution, supporting the presence of an underlying global construct. The average proportion of actual variance in the items explained by the factors was 71% in the bifactor solution compared to 64% in the first-order solution, indicating no decrease in reliability in the bifactor solution. Based on this, and considering the presence of a well-defined, highly reliable G-Factor and S-Factors retaining at least some specificity and acceptable levels of composite reliability, we considered that the bifactor model provided a better representation of the data (Morin et al., 2016). Noteworthy, the limited specificity of the interpersonal S-Factor when the variance explained by the G-Factor is considered supports the need for a bifactor representation of the data (Sánchez-Oliva et al., 2017). Altogether, the G-Factor provides a direct estimate of global infertility-related stress based on responses to all items. In contrast, the S-Factors represent specific components of infertility-related stress not already explained by the global component and unique to the intrapersonal and interpersonal subsets of items.

The bifactor model replicated well across Brazil and Italy, and multigroup analyses supported strict measurement invariance. Invariance of latent variance-covariance and latent means was also observed, indicating similar levels of inter-individual variability and latent factor scores across countries. Noteworthy, evidence of measurement invariance was established despite some differences in demographic (age and education) and infertility-related characteristics (infertility type and diagnosis) across Brazilian and Italian participants. Invariance of the IRSS bifactor model suggests that the coexistence of global infertility-related stress and its components represents a common pattern across Brazilian and Italian cultures. Brazilian and Italian infertile individuals thus seem to conceptualize and evaluate the burden of infertility in the same way (Steenkamp and Baumgartner, 1998). It is in line with the notion that infertility is a stress-triggering factor (Yazdani et al., 2017) that can affect the well-being and relationships of infertile people regardless of cultural differences (Stulhofer et al., 2013; Qadir et al., 2015). Brazilian and Italian participants in this study reported similar levels of infertility-related stress, both overall and in the intrapersonal and interpersonal domains. A previous study using a different self-report measure of infertility-related stress also reported no differences in mean scores between Brazilian and Italian infertile individuals (Gremigni et al., 2018).

As for the relations of IRSS-BP to sociodemographic and infertility-related characteristics, age, education, infertility type, and infertility diagnosis were unrelated to levels of infertility-related stress, in line with the IRSS original validation study (Casu and Gremigni, 2016) and other research in the Brazilian context (Casu et al., 2018, 2019), although contrary to other studies (Patel et al., 2016; Lakatos et al., 2017; Zaidouni et al., 2018). We found instead that IRSS-BP scores varied depending on gender and duration of infertility. Women showed slightly higher infertility-related stress than men in global IRSS-BP and intrapersonal and interpersonal domains. This finding is in line with previous research and literature reviews reporting that infertility is more distressing for women than men, and women experience greater infertility-related stress than their male counterparts (Greil et al., 2010; Ying et al., 2015; Chaves et al., 2019). It is likely due to cultural stereotypes and gender role expectations, which emphasize the centrality of motherhood and childbearing in women’s social function, especially in developing societies (Greil et al., 2010; Ying et al., 2015). Previous Brazilian studies using the IRSS also reported a link between female gender and higher global infertility-related stress (Casu et al., 2018, 2019). In the original validation study of the IRSS, Italian women reported a significantly higher level of infertility stress than men in the intrapersonal but not in the interpersonal domain (Casu and Gremigni, 2016). This discrepancy might be due to different sample characteristics. While participants in this study were seeking or already undergoing fertility treatment at the time of data collection, those in the Italian validation study were at their first visit to the fertility clinic. A gender gap has been observed in the diagnostic journey of the infertile couple, where the woman is the recipient of most diagnostic examinations (Gullo et al., 2021). Therefore, it is plausible that women in the Italian validation study were experiencing an acute stress reaction that characterizes initial stages involving the diagnostic work-up and mainly affects the intrapersonal life domain (Berg and Wilson, 1991).

We found that as the duration of the infertility problem increased, so did infertility-related stress. However, after more than 6 years of attempting conception, the levels of infertility-related stress tended to decrease, and there were no significant differences in the reported impact of infertility in the intrapersonal domain between individuals who had been trying to conceive for 1–2 years and those with more than 6 years of infertility. This is coherent with research indicating that a time frame of 3–6 years of infertility is characterized by the most significant risk for emotional maladjustment (Domar et al., 1992; Drosdzol and Skrzypulec, 2009). As previously suggested, during the first 1–2 years, infertile individuals tend to be optimistic about the possibility of conception, then begin to feel hopeless, and finally start to solve their feelings and accept remaining childless or turn to alternative parenthood options such as adoption (Domar et al., 1992; Adewunmi et al., 2012).

Our results also indicated that the perceived impact of infertility was significantly higher in the intrapersonal than in the interpersonal domain, as also reported in the IRSS original validation study (Casu and Gremigni, 2016) and coherent with previous evidence that the stressful implications of infertility concern the domain of self, more than social life (Karabulut et al., 2013; Swanson and Braverman, 2021). In this study, the difference between levels of infertility-related stress in the two life domains was moderate for a duration of infertility of 1–2 years and small for more than 2 years of infertility duration. Specifically, scores in the interpersonal domain were higher as the duration of infertility increased. This is coherent with previous findings that a longer duration of infertility was associated with greater infertility-related stress in the social areas of family and social relationships and work-life (Karabulut et al., 2013; Keramat et al., 2014; Santoro et al., 2016). However, the fertility treatment stage and number of previous treatment cycles might also be associated with infertility-related stress changes in both domains (Volpini et al., 2020). Therefore, prospective studies should be conducted to examine whether changes in IRSS-BP scores are a response to the duration of infertility or the duration of infertility treatments.

As for relations to depressive symptomatology, regardless of gender, participants with above-threshold levels of depression showed higher total and subscale IRSS-BP scores than participants with below-threshold depression. Differences among the BDI-II groups were large in the intrapersonal domain, medium-to-large in global infertility-related stress, and moderate in the interpersonal domain. These results are coherent with those reported in the Italian validation study (Casu and Gremigni, 2016), where highly depressed women and men had significantly higher scores than non-depressed ones in both IRSS components, with a more substantial effect in the intrapersonal than the interpersonal domain. In this study, bivariate correlations with BDI-II scores for global IRSS-BP and the interpersonal domain were strong and moderate, respectively, among women but non-significant among men. In contrast, those for the intrapersonal domain were strong among women and small-to-moderate among men. Differently, in the Italian validation study, the correlations of BDI-II with global and intrapersonal infertility-related stress were strong for men and moderate for women, and those with the interpersonal IRSS domain were moderate for both genders. Such a discrepancy might be due to cultural differences, as Brazil has a more distinct patriarchal culture and collectivist features than Italy (Oyserman and Sorensen, 2009). Previous research on Brazilian infertile individuals indeed indicated that Brazilian women, compared to their male counterparts, had more negative emotional reactions to interpersonal aspects such as being questioned about childlessness by relatives and friends and being invited to a child’s birthday party (Franco et al., 2002). Also, the correlations observed in this study are coherent with evidence that the associations of depression with infertility-related stress were stronger for women than for men (Chaves et al., 2019), and those with stress in the social areas of life were significant for women but not for men (Peterson et al., 2003), thus supporting the concurrent validity of the IRSS-BP.

### Limitations and Future Directions

The present study has some limitations. First, using a convenience sample and the collection of Brazilian data in one clinical setting only limit the generalizability of results. Second, reliability was assessed as model-based internal consistency only; future studies should therefore assess test–retest stability as well. Third, the performance of the IRSS-BP was not compared with other measures of the same construct, such as the FPI (Newton et al., 1999) and the FertiQoL (Boivin et al., 2011), and associations with other psychological variables were limited to levels of depression. Due to the small size of the subsample that completed the BDI-II, we could not examine the IRSS-BP nomological validity by integrating a BDI-II latent factor in the bifactor ESEM measurement model. This would allow for a simultaneous examination of the associations of global and specific IRSS-BP factors with the external criterion variables to clarify whether the IRSS-BP S-Factors have sufficient specificity to result in significant relations with the criteria over and above the prediction provided by the G-Factor. Future studies with larger samples should test for the validity of the IRSS-BP bifactor model within a semantic network of similar or different constructs. Fourth, other individual characteristics and infertility conditions that we have not measured could influence the individual’s infertility-related stress; therefore, a more extensive collection of sociodemographic and clinical information should be done in future research. Finally, the sample was composed of independent individuals, although infertility-related stress is a shared experience within a couple (Pasch and Sullivan, 2017). Thus, future studies could collect data from both couple members and use a dyadic approach to test for dyadic invariance of the IRSS-BP bifactor model across partners of the infertile couple (Claxton et al., 2015).

## Conclusion

Our findings indicated that the underlying structure of IRSS-BP scores was best represented by a bifactor solution incorporating an overarching infertility-related stress factor and two specific components of intrapersonal and interpersonal life domains affected by infertility stress. Both the general and the specific IRSS-BP factors showed adequate levels of composite reliability and validity evidence based on relations to sociodemographic and infertility-related characteristics and depressive symptomatology. Therefore, when using the IRSS-BP in research and clinical practice, we suggest considering the total score and the two subscale scores of intrapersonal and interpersonal infertility-related stress.

Altogether, the present study provides initial evidence of validity and reliability for the Brazilian-Portuguese language version of the IRSS to be used in the Brazilian context to rapidly assess the burden of infertility at both global and domain-specific levels. The IRSS-BP could be used by staff in fertility clinics to identify patients who need support to deal with the stressful impact of infertility on the areas of the self and social life, as recommended by international guidelines for routine psychosocial care as a means to improve infertile patients’ well-being and compliance with treatment (Gameiro et al., 2015).

## Data Availability Statement

The raw data supporting the conclusions of this article are available on request from the corresponding author.

## Ethics Statement

The studies involving human participants were reviewed and approved by Ethics Committee of Faculdade de Medicina do ABC and Ethics Committee of University of Bologna. The patients/participants provided their written informed consent to participate in this study.

## Author Contributions

GC: data analysis and interpretation, software, visualization, writing-original draft, and writing-review and editing. VZ: study concept and design, provision of study materials and patients, collection and assembly of data, data analysis, writing-original draft, and writing-review and editing. AP: study concept and design, provision of study materials and patients, writing review and editing. EM and BB: collection and assembly of data, and writing-original draft. CB: writing-original draft. PG: study concept and design, provision of study materials, supervision, and writing–review and editing. All authors contributed to the manuscript and approved the final version.

## Conflict of Interest

The authors declare that the research was conducted in the absence of any commercial or financial relationships that could be construed as a potential conflict of interest.

## Publisher’s Note

All claims expressed in this article are solely those of the authors and do not necessarily represent those of their affiliated organizations, or those of the publisher, the editors and the reviewers. Any product that may be evaluated in this article, or claim that may be made by its manufacturer, is not guaranteed or endorsed by the publisher.
