# Supplementary material for: The Infertility-Related Stress Scale: Validation of a Brazilian–Portuguese Version and Measurement Invariance Across Brazil and Italy
Source: Front Psychol. 2022 Jan 13;12:784222. doi: 10.3389/fpsyg.2021.784222 (PMC8792459; doi:10.3389/fpsyg.2021.784222)
Supplement: Supplementary file 1 [file Data_Sheet_1.docx]

Supplementary Material

# Table S1. Descriptive statistics of IRSS-BP items (*n* = 553).

| **Item** | ***M*** | ***SD*** | ***Sk*** | ***K*** | **Gender** | **Age** |
| --- | --- | --- | --- | --- | --- | --- |
| 1. Physical well-being | 2.61 | 1.77 | 0.78 | -0.48 | -0.15^***^ | -0.01 |
| 2. Relatives | 2.64 | 1.73 | 0.86 | -0.17 | -0.12^**^ | -0.02 |
| 3. In-laws | 2.71 | 1.84 | 0.81 | -0.43 | -0.12^**^ | -0.04 |
| 4. Leisure and enjoyment | 2.19 | 1.57 | 1.15 | -0.40 | -0.17^***^ | -0.07 |
| 5. Marital satisfaction | 3.28 | 1.82 | 0.31 | -0.92 | -0.07 | -0.06 |
| 6. Mental well-being | 3.18 | 1.87 | 0.46 | -0.83 | -0.21^***^ | -0.11^**^ |
| 7. Performance at work/housework | 2.39 | 1.69 | 0.99 | 0.01 | -0.14^***^ | -0.04 |
| 8. Close friends | 2.40 | 1.66 | 1.00 | 0.12 | -0.13^**^ | -0.03 |
| 9. Sexual pleasure | 2.91 | 1.93 | 0.61 | -0.77 | -0.11^**^ | -0.05 |
| 10. Colleagues | 2.30 | 1.66 | 1.07 | 0.15 | -0.15^***^ | -0.01 |
| 11. Neighbors | 2.07 | 1.59 | 1.39 | 0.99 | -0.10^*^ | -0.02 |
| 12. Global life satisfaction | 3.41 | 1.94 | 0.32 | -1.01 | -0.18^***^ | -0.09^*^ |

*Sk*, skewness; *K*, kurtosis; standard error for *Sk* = 0.10; standard error for *K* = 0.21; point-biserial correlations are displayed for gender (coded as 0 = woman and 1 = man); product-moment Pearson’s correlations are displayed for age.

^*^ *p* < 0.05, ^**^ *p* ≤ 0.01, ^***^ *p* ≤ 0.001.

# Table S2. Item-level components of variance in the first-order and bifactor ESEM solutions.

|  | **First-order ESEM** | | | | **Bifactor ESEM** | | | | |
| --- | --- | --- | --- | --- | --- | --- | --- | --- | --- |
| **Item** | **σ^2^_error_** | **σ^2^_true_**  **_(Factor)_** | **σ^2^_true_**  **_(Cross-loading)_** | **σ^2^_true_**  **_(Total)_** | **σ^2^_error_** | **σ^2^_true_**  **_(G-Factor)_** | **σ^2^_true_**  **_(S-Factor)_** | **σ^2^_true_**  **_(Cross-loading)_** | **σ^2^_true_**  **_(Total)_** |
| 1. Physical well-being | 0.345 | 0.510 | 0.016 | 0.526 | 0.351 | 0.432 | 0.217 | 0.001 | 0.650 |
| 2. Relatives | 0.387 | 0.347 | 0.060 | 0.407 | 0.104 | 0.832 | 0.064 | 0.001 | 0.897 |
| 3. In-laws | 0.460 | 0.333 | 0.040 | 0.373 | 0.266 | 0.701 | 0.032 | 0.000 | 0.733 |
| 4. Leisure and enjoyment | 0.461 | 0.198 | 0.120 | 0.318 | 0.468 | 0.428 | 0.091 | 0.013 | 0.532 |
| 5. Marital satisfaction | 0.514 | 0.381 | 0.011 | 0.392 | 0.486 | 0.379 | 0.116 | 0.017 | 0.512 |
| 6. Mental well-being | 0.186 | 0.922 | 0.007 | 0.929 | 0.172 | 0.397 | 0.430 | 0.000 | 0.827 |
| 7. Performance at work/housework | 0.354 | 0.356 | 0.067 | 0.423 | 0.355 | 0.594 | 0.025 | 0.026 | 0.645 |
| 8. Close friends | 0.131 | 0.933 | 0.002 | 0.935 | 0.143 | 0.771 | 0.085 | 0.000 | 0.856 |
| 9. Sexual pleasure | 0.393 | 0.566 | 0.001 | 0.567 | 0.400 | 0.371 | 0.228 | 0.002 | 0.601 |
| 10. Colleagues | 0.152 | 0.990 | 0.012 | 1.002 | 0.114 | 0.701 | 0.184 | 0.001 | 0.886 |
| 11. Neighbors | 0.341 | 0.835 | 0.023 | 0.858 | 0.347 | 0.539 | 0.110 | 0.004 | 0.653 |
| 12. Global life satisfaction | 0.244 | 0.903 | 0.014 | 0.917 | 0.232 | 0.345 | 0.423 | 0.000 | 0.768 |

*σ^2^*_error_, uniqueness (*δ*), indicating the proportion of variance in item ratings due to error variance; *σ^2^*_true_, squared loading (*λ^2^*), indicating the proportion of variance in item ratings due to true score variance.

**
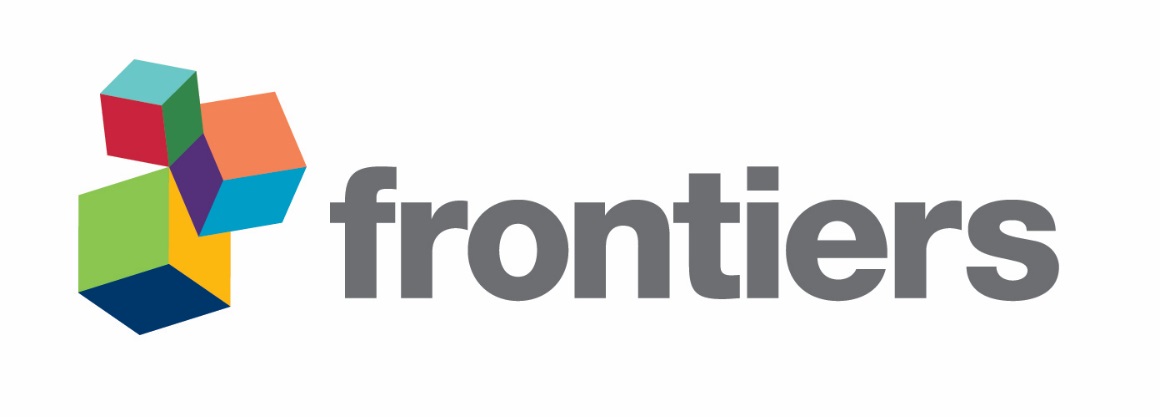
**
